# Improvements of tooth movement efficiency and torque control in expanding the arch with clear aligners: a finite element analysis

**DOI:** 10.3389/fbioe.2023.1120535

**Published:** 2023-06-01

**Authors:** Song Yao, Wei Jiang, Chunjuan Wang, Yao He, Chao Wang, Lan Huang

**Affiliations:** ^1^ Stomatological Hospital of Chongqing Medical University, Chongqing, China; ^2^ Chongqing Key Laboratory of Oral Diseases and Biomedical Sciences, Chongqing, China; ^3^ Chongqing Municipal Key Laboratory of Oral Biomedical Engineering of Higher Education, Chongqing, China; ^4^ Key Laboratory of Biomechanics and Mechanobiology, Ministry of Education, Beijing Advanced Innovation Center for Biomedical Engineering, School of Biological Science and Medical Engineering, School of Engineering Medicine, Beihang University, Beijing, China

**Keywords:** finite element analysis, clear aligner, arch expansion, tooth movement strategies, torque compensation

## Abstract

**Objectives**: The purpose of this study was to analyze the effect of different movement strategies, embossment structures, and torque compensation of the aligner on tooth movement during arch expansion using clear aligners by finite element analysis.

**Methods:** Models comprising the maxilla, dentition, periodontal ligament, and aligners were created and imported into a finite element analysis software. The tests were performed using the following: three orders of tooth movement (including alternating movement with the first premolar and first molar, whole movement with second premolar and first molar or premolars and first molar), four different shapes of embossment structures (ball, double ball, cuboid, cylinder, with 0.05, 0.1, 0.15-mm interference) and torque compensation (0°, 1°, 2°, 3°, 4°, and 5°).

**Results:** The expansion of clear aligners caused the target tooth to move obliquely. Alternating movement resulted in higher movement efficiency with lower anchorage loss as compared with whole movement. Embossment increased the efficiency of crown movement but did not contribute positively to torque control. As the angle of compensation increased, the tendency of oblique tooth movement was gradually controlled; however, the movement efficiency decreased concurrently, and stress distribution on the periodontal ligament became more even. For each 1° increase in compensation, the torque per millimeter of the first premolar would decrease by 0.26°/mm, and the crown movement efficiency eliminate decreased by 4.32%.

**Conclusion:** Alternating movement increases the efficiency of the arch expansion by the aligner and reduces anchorage loss. Torque compensation should be designed to enhance torque control in arch expansion using an aligner.

## 1 Introduction

Clear aligner therapy (CAT) is becoming increasingly popular owing to its esthetic, clean, and comfortable features, which are preferred by clinicians and patients (Shokeen et al., 2022). CAT divides the tooth movement process into a series of tiny sequential movements that ultimately help achieve the targeted movement of the tooth. The slight offset of the clear aligner from the dentition creates an elastic force that further applies an orthodontic force to the tooth, causing it to move towards the target position (Cortona et al., 2020). Currently, CAT is available for a wide range of tooth movements, such as intrusion (Liu et al., 2021), molar distalization (Ji et al., 2022), and root torque (Liu et al., 2022), expanding clinical opportunities to treat complex orthodontic cases (Inchingolo et al., 2022).

Insufficient width of the maxillary arch results in narrowing of the dentition and a molar crossbite, which will then affect occlusal function and esthetics (Lee, 1999). Many studies have confirmed the ability of CAT to expand the arch; however, the actual efficiency of CAT is lower than the preset value (Solano-Mendoza et al., 2017) and decreases gradually by moving from the premolar to molar (Houle et al., 2017; Lione et al., 2021). A quantitative analysis of a change in torque of arch expansion using CAT is yet to be reported. Moreover, the type of tooth movement in CAT tends to be buccal tilt (Houle et al., 2017), and a less predictable buccal tilt is associated with a risk of severe periodontal destruction, including buccal gingival recession (Morris et al., 2017) and bone dehiscence (Sulewska et al., 2021). Therefore, it is worthwhile to further investigate how CAT can improve tooth movement efficiency and torque control.

Finite element analysis (FEA) is a computer simulation technique that can be used for complex biomechanical analyses. The advantages of this technique include the ability to create many different simulations with tightly controlled variables and high reproducibility, to provide an appropriate database for future clinical trials, helping to avoid unnecessary duplication of clinical trials (Romanyk et al., 2020; Liu et al., 2022).

Therefore, the aim of this paper is to evaluate the effect of different tooth movement strategies on movement efficiency during arch expansion using CAT and to compare the effects of different embossment designs and torque compensation on movement efficiency and torque control by FEA (Supplementary Table S1).

## 2 Materials and methods

### 2.1 Construction of an orthodontic model

**FIGURE 1:**
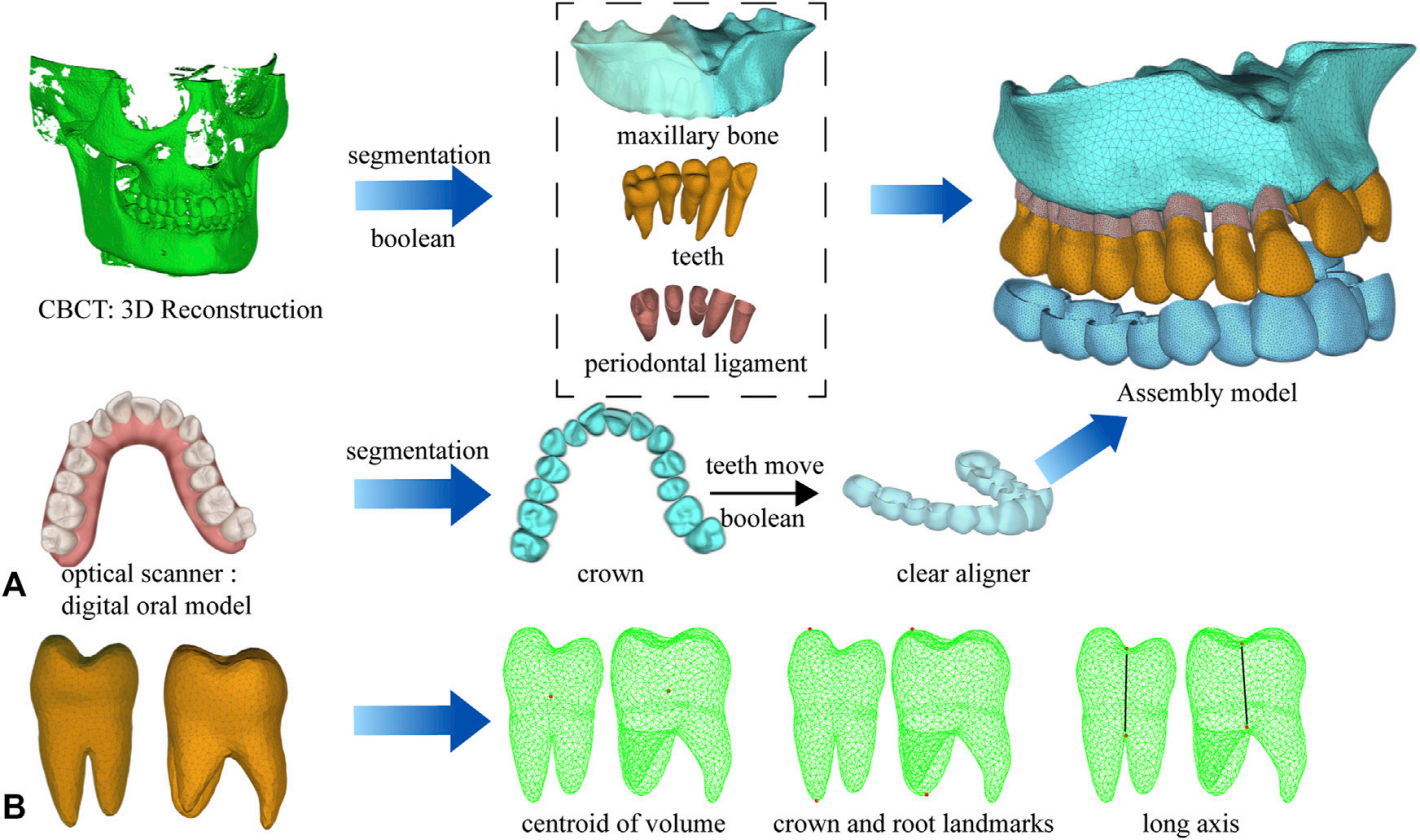
Computer-aided design model and measurement points. **(A)** The process of model building and assembly. **(B)** Measurement points of tooth movement distance and torque change.

This study was approved by the Stomatological Hospital of Chongqing Medical University Ethics Committee (NO. 2022-038). The study participant was a healthy adult orthodontic patient with a narrow dental arch. For starters, the maxilla and dentition data of the participant were acquired using cone-beam computed tomography (CBCT, Kava, Biberach, Germany) and intraoral scanning (iTero, Invisalign, United States), and three-dimensional models of the maxilla and dentition were constructed (Barone et al., 2017) using Mimics Research (19.0; Materialise, Leuven, Belgium), 3-Matic (11.0; Materialise, Leuven, Belgium), and Geomagic Warp (3D systems, Rock Hill, SC) software. Additionally, the right maxilla and dentition were mirrored to establish a symmetric model to improve the efficiency of the subsequent FEA and guarantee a realistic model (Jia et al., 2022). After having established a symmetric model, each tooth was offset along the normal line outward by 0.2 mm (Seo et al., 2021). Moreover, through a Boolean operation, the PDL, also known as periodontal ligament model was acquired (Liu et al., 2022). After having obtained the PDL, we were able to obtain the maxillary alveolar socket by subtracting the teeth and PDL models from the maxilla using Boolean operations (Supplementary Figure S1). Finally, the maxillary, PDL, dentition, and clear aligner models were assembled in 3-Matic software to produce an FEA solid model with unstructured 4-noded tetrahedral elements in Figure 1A. Specifically, the dimensions of the mesh divisions were 1.0 mm for the tooth, 2.0 mm for the maxilla, 0.2 mm for the PDL, and 0.5 mm for the aligners. The maxilla, teeth, PDL, and aligners were set as linearly elastic (Kojima and Fukui, 2012). The material properties (Gomez et al., 2015) and the approximate number of nodes and mesh are listed in Table 1. Following, these operations were completed using the MIMICS, 3-Matic and ABAQUS (2020; SIMULIA, Providence, RI) software.

**TABLE 1:** Material properties and number of nodes and elements of the components of the finite element model.

Component	Young’s modulus (MPa)	Poisson’s ratio	Nodes	Elements
Teeth	18,600	0.31	178752	123439
PDL	0.68	0.49	152139	76970
Bone	13,700	0.3	137197	81737
Clear aligner	816.31	0.3	313998–342416	203691–224513

### 2.2 Design of aligner model

#### 2.2.1 Order of teeth movement

**FIGURE 2:**
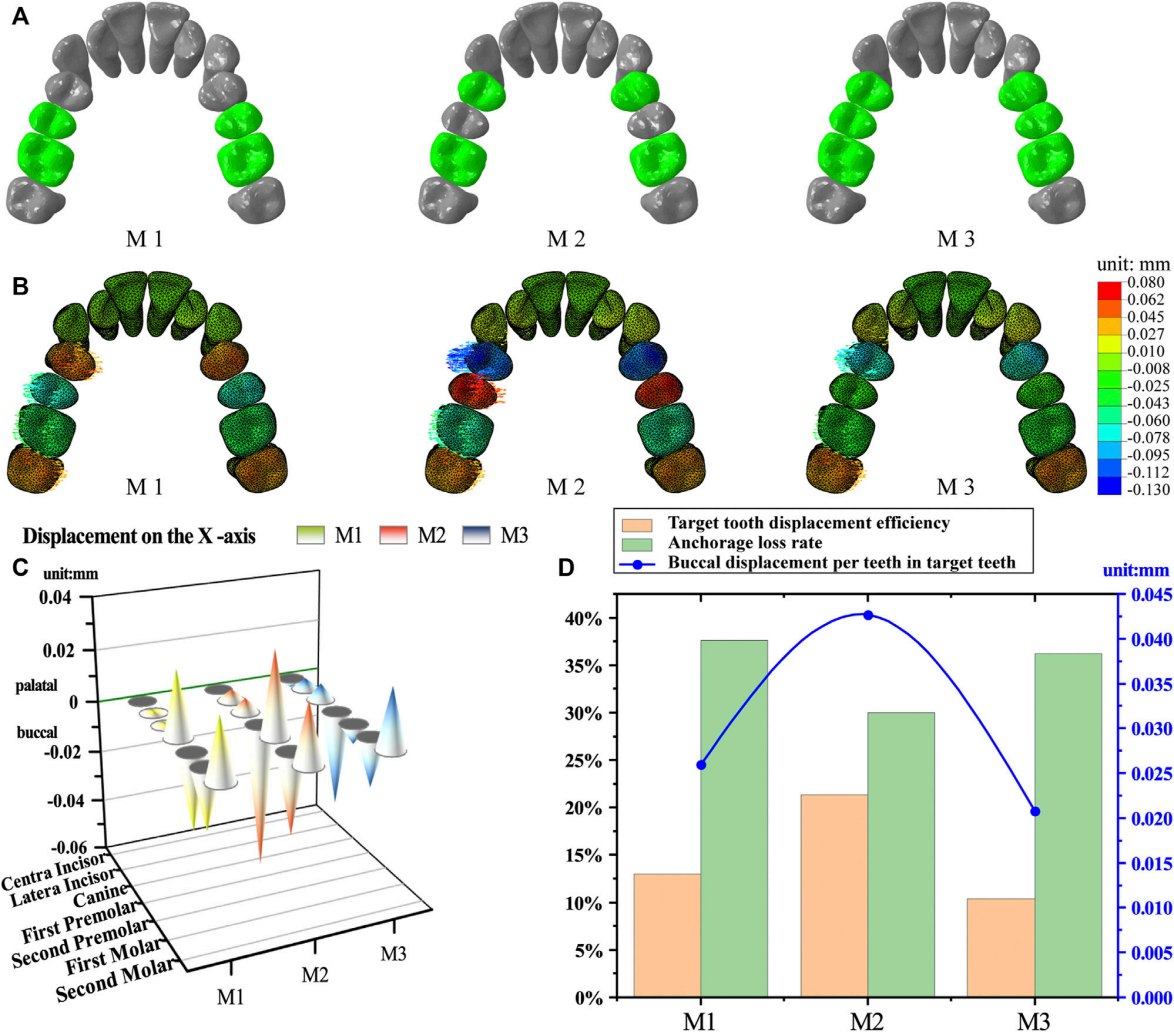
Tooth movement strategies. **(A)** Grouping of movement strategies. M1, moving second premolar and first molar alternative movement. M2, moving first premolar and first molar. M3, moving premolars and first molar. M1 and M3 for whole movement, M2 for alternating movement. **(B)** The initial displacements of the teeth. **(C)** Buccolingual displacement of the teeth. **(D)** Target tooth displacement efficiency, anchorage loss rate and buccal moving distance per teeth in target teeth.

We selected several different tooth movement patterns for expanding the arch. The optimal tooth movement strategy was filtered based on the movement efficiency of the target teeth and the loss of anchors. The strategies of the different tooth movement are the following: M1 moves the second premolar and first molar. M2 moves the first premolar and first molar. M3 moves premolars and the first molar. The target teeth are moved buccally by 0.2 mm. We refer to the intraoral scanning to trim the dentition along the gingival margin, mark it as the inner surface of clear aligner, and then offset by 0.75 mm (Xia et al., 2022) outward according to the surface normal direction to generate the clear aligner model (Figure 2A). M2 represents alternating movement, M1 and M3 represent whole movement.

#### 2.2.2 Embossment of aligner

**FIGURE 3:**
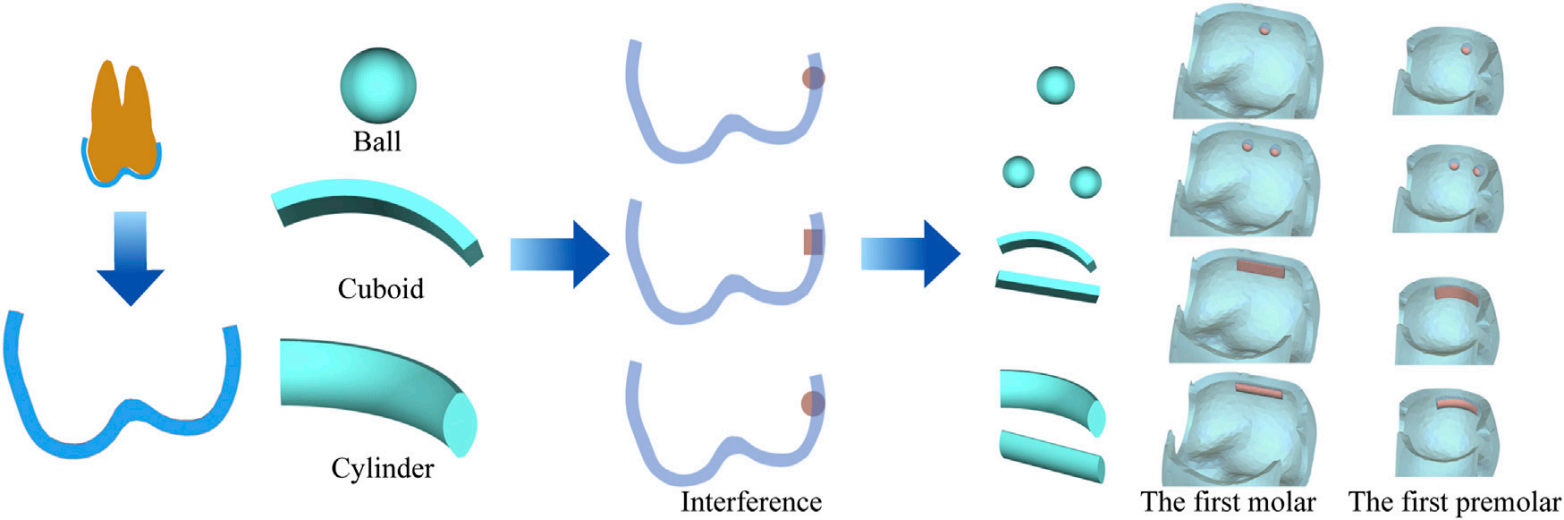
Embossment of aligner, including ball (diameter: 0.8 mm, 0.85 mm, 0.90 mm), double-ball (diameter: 0.90 mm), cuboid (3.5*1*0.85 mm, 3.5*1*0.9 mm), cylinder (3.5*1*0.85 mm, 3.5*1*0.9 mm). The interference (0.05, 0.10, and 0.15 mm) is equal to the difference between the thickness of the auxiliary shapes and the aligner (0.75 mm).

For starters, the effects of different embossments on tooth movement, including movement efficiency and torque control, were measured. The structure of the embossment in the aligner, that we measured is formed by means of aligner and auxiliary shapes (Figure 3). Moreover, we added auxiliary shapes on the inner surface of the aligner at a site corresponding to the cervical portion of the palatal surface of the crown, including balls, double balls, cuboids, and cylinders. Once we added the auxiliary shapes, the aligner no longer fit tightly to the crown surface, and the embossment generated an interference contact, which simulated a buccal push to the tooth. Subsequently we designed a buccal movement of 0.2 mm for the first premolar and first molar to create an aligner with no embossment as a blank control (this strategy had shown higher movement efficiency in the previous part of the experiment and was therefore used as a blank control in this part, as is explained in the Results section). Based on the blank control, the aforementioned embossment structures were added to the aligners. The experimental groups were named according to the extent of interference and shape of the embossment.

#### 2.2.3 Torque compensation of aligner

We designed different torque compensations (*θ* = 0°, 1°, 2°, 3°, 4°, and 5°) based on a 0.2 mm buccal shift of the target tooth to measure the tooth torque generated when the aligner expanded the arch. Torque compensation was achieved by rotating the target tooth anticlockwise (crown to the palatal side and root to the buccal side) at the center of the tooth volume and then designing the aligner with the repositioned dentition.

### 2.3 Coordinate system setting

A global coordinate system was used to define the orientation of the x-, y-, and *z*-axes. The *x*-axis represented the intersection of the coronal and occlusal planes. The positive direction pointed to the left side of the patient. The *y*-axis was perpendicular to the *x*-axis, with the positive direction pointing posteriorly. The *z*-axis was perpendicular to the x- and *y*-axes, with the positive direction pointing apically.

### 2.4 Measurement and analysis

The initial displacement tendencies of the target and anchorage teeth and the equivalent stress of the PDL were measured. The anchor loss rate (Figure 2D) was used to evaluate the efficiency of different movement strategies, which is shown in the following equations. We further describe the movement of the target teeth based on the target tooth displacement efficiency (the ratio of the actual value of the sum of the buccal positions of the target teeth to the present value) and the buccal moving distance per target tooth.

$$The\;anchor\;loss\;rate=\frac{Tooth-average\;anchor\;loss}{Tooth-average\;arch\;expansion}$$

$$Tooth-average\;anchor\;loss=\frac{Sum\;of\;the\;buccolingual\;displacement\;of\;the\;anchorage\;teeth}{The\;number\;of\;anchorage\;teeth\;during\;arch\;expansion}$$

$$Tooth-average\;arch\;expansion=\frac{Sum\;of\;the\;buccal\;displacement\;of\;the\;target\;teeth}{The\;number\;of\;target\;teeth\;during\;arch\;expansion}$$

The centroid of the tooth volume, buccal cusp, and buccal root were used as measurement points to observe tooth movement. The angular variation of the long axis of the tooth in the coronal plane was measured to evaluate torque change (Figure 1B).

## 3 Results

### 3.1 Alternating movement is more efficient than whole movement

The target teeth moved buccally, and the posterior anchored teeth moved palatally with all strategies (M1, M2, and M3). The cuspids and incisors tended to move with a labial tilt, but this movement was significantly less than that of the posterior teeth (Figure 2B, Supplementary Videos S1–S3).

The M2 group showed the greatest buccal displacement of the target teeth as well as the greatest undesirable palatal displacement of the anchorage teeth in all groups. Although the second premolar in the M3 group moved buccally as planned, its displacement was one order of magnitude lower than that of the other target teeth (Figure 2C; Table 2).

**TABLE 2:** *X*-axis movement per tooth in group M1, M2, and M3 (unit: mm).

Group	Central incisor	Lateral incisor	Canine	First premolar	Second premolar	First molar	Second molar
M1	−0.00038	0.000785	0.000587	0.02508	−0.02907	−0.02288	0.022754
M2	−0.00058	0.004576	0.005551	−0.05474	0.031878	−0.03071	0.02259
M3	−0.0035	0.004349	0.006471	−0.03603	−0.0076	−0.01865	0.02278

*X*-axis, posterior teeth are buccolingual (buccal side is negative direction), anterior teeth are proximo-distal (distal side is negative direction). Target teeth of M1 group are the second premolar and first molar. Target teeth of M2 group are first premolar and first molar. Target teeth of M3 group are premolars and first molar.

As shown in Figure 2D, the M2 group had the highest target tooth displacement efficiency (21.35%), which was greater than that in M1 (13%) and M3 (10.38%) groups, while the M2 group showed the greatest buccal movement per tooth in the target teeth (0.0427 mm), which was greater than that in the M1 (0.026 mm) and M3 (0.0208 mm) groups. The lowest anchorage loss rate was found in the M2 group (30.02%), followed by M3 (36.24%) and M1 (37.62%) groups.

### 3.2 Embossment increases efficiency based on the excessive tilting movement

**FIGURE 4:**
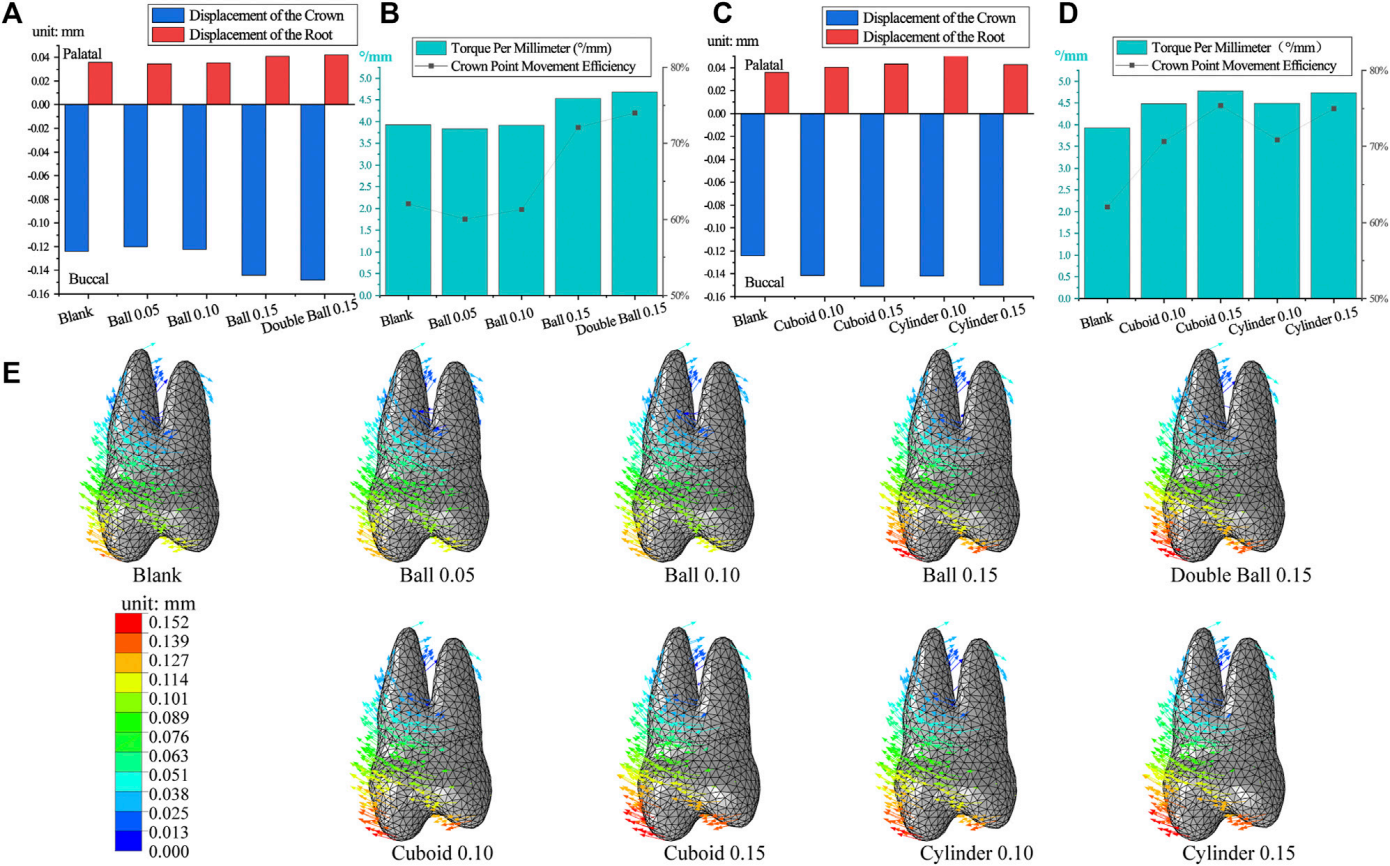
The movement of the first premolar in embossment groups. **(A,C)** The crown-root movement in embossment groups. **(B,D)** The torque per millimeter and crown movement efficiency in embossment groups. **(E)** The displacement tendencies of the first premolar in embossment groups.

**FIGURE 5:**
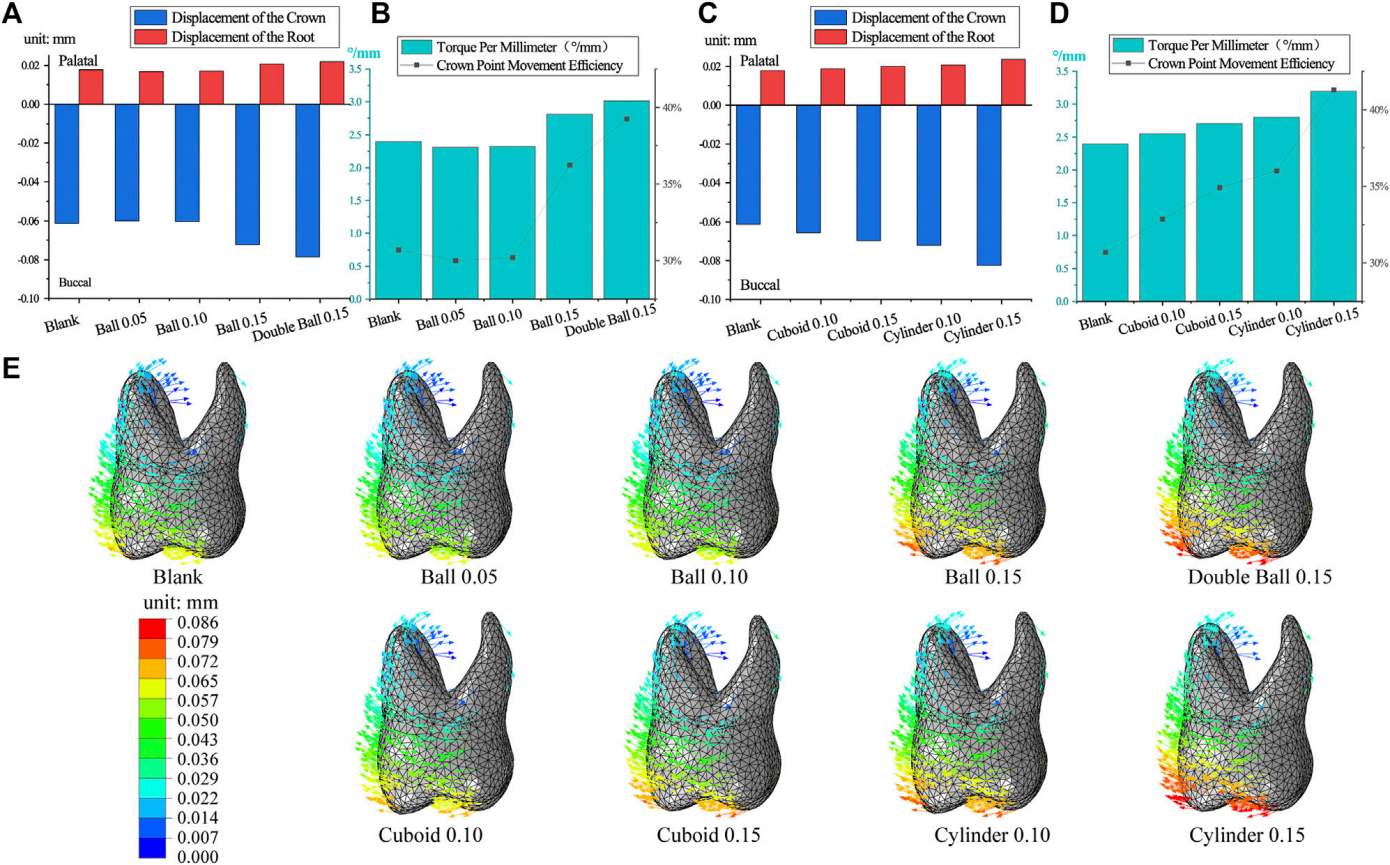
The movement of the first molar in embossment groups. **(A,C)** The crown-root movement in embossment groups. **(B,D)** Torque per millimeter and crown movement efficiency in embossment groups. **(E)**, displacement tendencies of the first molar in embossment groups.

As the diameter of the ball-like embossment increased, the crown-root movement of both first premolar and first molar increased. However, the tooth movement was oblique (Figures 4A, E), and the crown point movement efficiency and torque per millimeter also increased (Figure 4B). This trend previously explained, was more prominent in the double-ball group than in the ball group, with the same interference. Crown point movement efficiency of the first premolar in the blank group was 62.05% and the torque per millimeter was 3.93°/mm, which was greater than those in the ball 0.05 and 0.1 interference groups but less than those in the ball 0.15 and double ball 0.15 interference groups (Figure 4B). Equally important, the movement of the first molar was similar, and the crown point movement efficiency of first molar in the blank group was 30.7% and torque per millimeter was 2.39°/mm (Figures 5A, B, E).

Cuboid and cylinder groups were also similar because as interference increased, the crown-root movement increased for both the first premolar and first molar (Figures 4C, E, 5C, E), the crown point movement efficiency and torque per millimeter also increased along with the tendency of oblique movement (Figure 4D; Figure 5D). The performance of the first premolar in the cylinder group was similar to that in the cuboid group, but the measurements of the first molar in the cylinder group were greater than those in the cuboid group (cylinder 0.15> cylinder 0.1> cuboid 0.15> cuboid 0.1) (Figure 5D).

### 3.3 Torque compensation reduces the tendency of tilting

**FIGURE 6:**
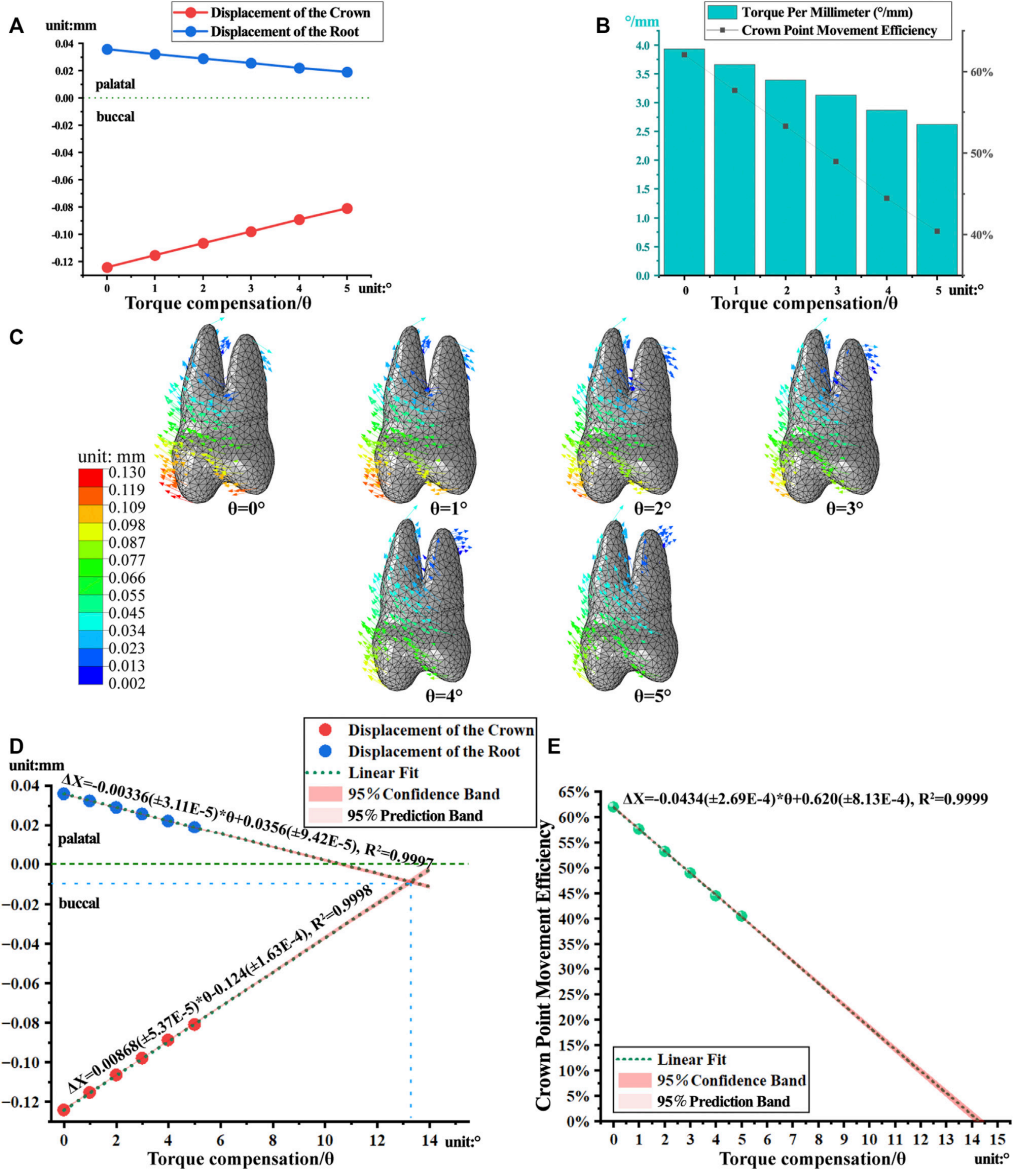
The movement and linear regression model of the first premolar with torque compensation. **(A)** The crown-root displacement with torque compensation. **(B)** The torque per millimeter and crown movement efficiency. **(C)** The displacement tendencies of the first premolar with torque compensation. **(D, E)** The linear regression model of crown-root displacement and torque per millimeter.

As the angle of compensation increased, the crown-root point movement of the first premolar decreased. Similarly, the torque per millimeter and crown point movement efficiency also decreased. Torque per millimeter of the first premolar decreased by 0.26°/mm, and crown point movement efficiency decreased by 4.32% for approximately every 1° increase, within 1°–5° of the compensation angle (Figures 6A, B). As the angle increased, buccal movement of the first premolar decreased, and the changes of the palatal root changed from extrusion to intrusion (Figure 6C).

Based on a linear regression model (R^2^ > 0.99) matched to the measured data, it was predicted that when the angle of compensation is approximately 13°, the crown and root of first premolar are displaced equally and towards the buccal side, achieving bodily movement (Figure 6D). However, the predicted result of crown point movement efficiency dropped almost to 5.6% (Figure 6E).

**FIGURE 7:**
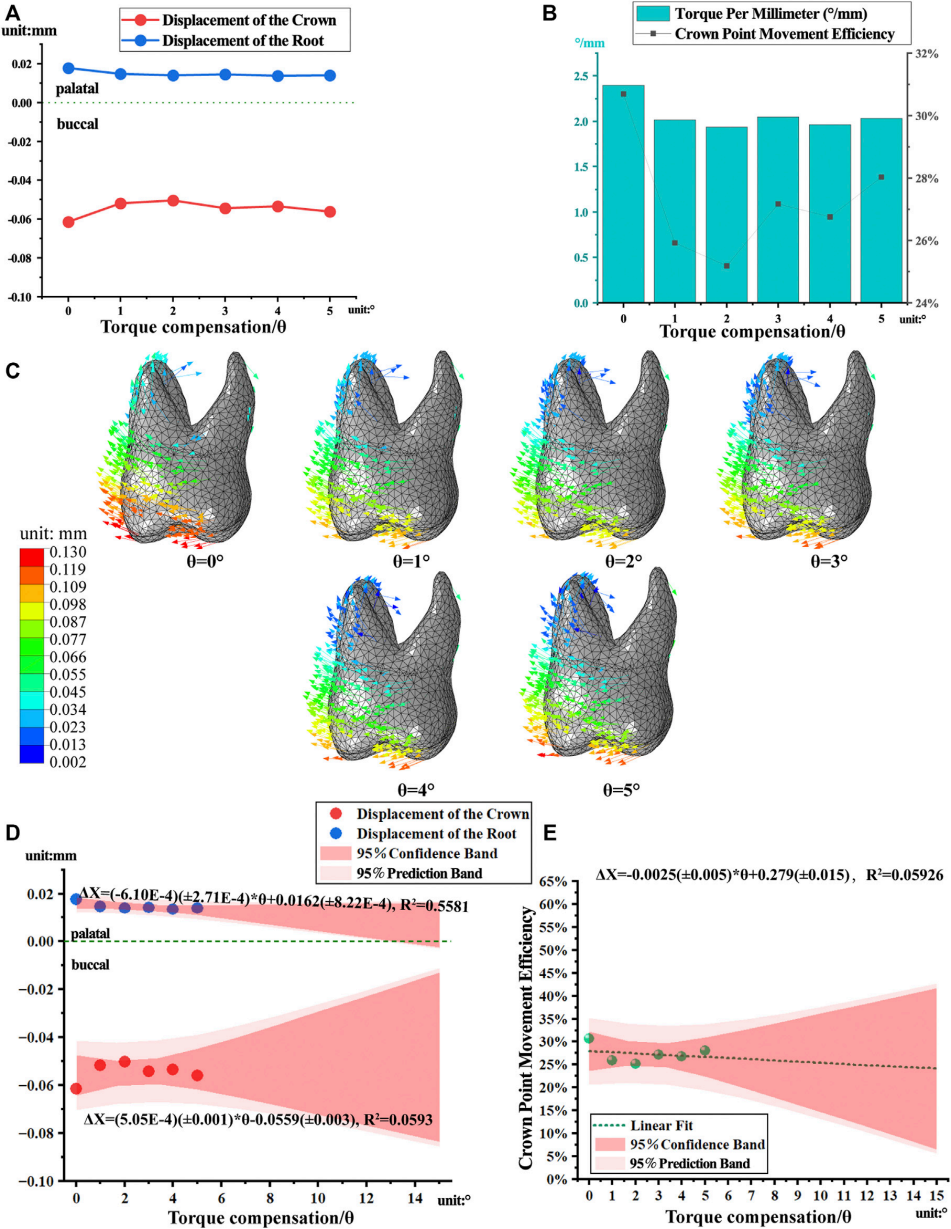
The movement and linear regression model of the first molar with torque compensation. **(A)** The crown-root displacement with torque compensation. **(B)** The torque per millimeter and crown movement efficiency. **(C)** The displacement tendencies of the first molar with torque compensation. **(D, E)** The linear regression model of crown-root displacement and torque per millimeter.

Torque compensation reduced the torque per millimeter of molar but lacked predictability. As the angle of compensation increased, the torque per millimeter and crown point movement efficiency of the first molar decreased (Figures 7A, B). However, the above data did not show linear relationships, as in the case of the first premolar. The first molar showed minimum torque per millimeter at 2° compensation, within 1–5° compensation. As compensation increased, the tendency of the crown to move buccally decreased, the magnitude of buccal root intrusion decreased, and the tendency of the palatal root to extrude palatally did not change significantly (Figure 7C).

Owing to the poor linear correlation of the first molar movement data, the regression model had a lower prediction reliability (Figures 7D, E).

**FIGURE 8:**
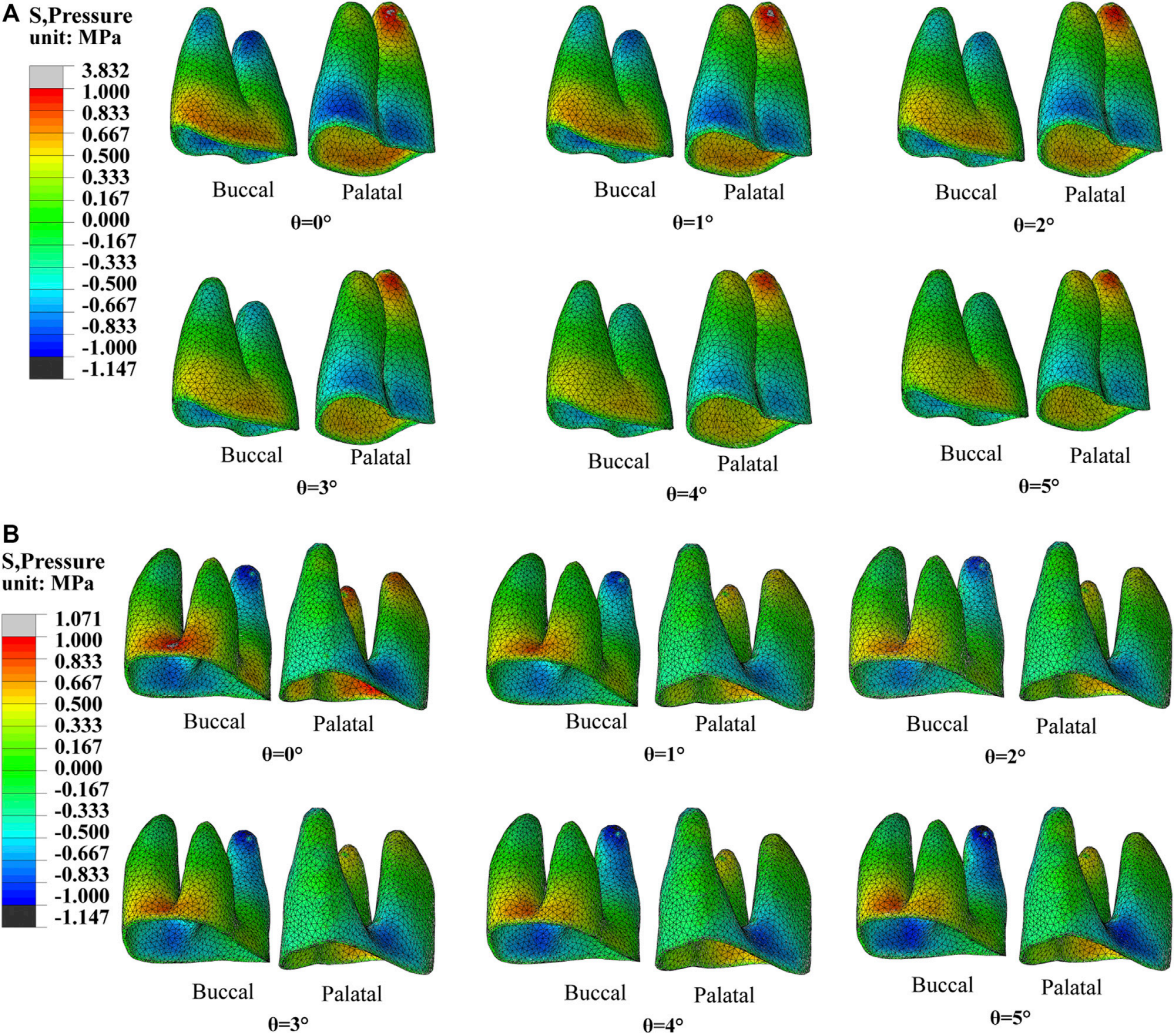
The equivalent stress of PDL with different torque compensation. **(A)** PDL of the first premolar. **(B)** PDL of the first molar.

Orthodontic force induces remodeling of periodontal tissues, resulting in bone resorption on the compressive side and bone deposition on the tensile side. Therefore, the distribution of compressive and tensile stresses on the surface of the periodontal ligament is utilized as an observational indicator of periodontal tissue remodeling and as an auxiliary predictor of tooth movement trends. For the first premolar and first molar with no torque compensation, aligners produced compressive stress in the buccal root neck and palatal root apex and tensile stress in the palatal root neck and buccal root apex. As the torque compensation increased, the equivalent stress was more evenly distributed over the PDL (Figure 8).

## 4 Discussion

The comfort and esthetic properties of CAT deserve recognition; however, numerous studies (Houle et al., 2017; Zhou and Guo, 2020) have found that CAT exhibits lower efficiency and predominantly utilizes tipping movements during arch expansion. Consequently, our research investigates the biomechanical effects of movement strategies, embossment, and torque compensation designs in CAT to enhance the efficiency and anchorage control of aligners. The results reveal that alternating movements outperform whole movements in terms of movement efficiency, while torque compensation demonstrates superior torque control compared to embossment.

We proposed an anchorage loss rate based on target and anchorage teeth displacement to evaluate different movement strategies. Among all groups, alternating movement M2 demonstrated the lowest anchorage loss rate, indicating its effectiveness in achieving target tooth displacement with minimal anchorage loss.

The CAT-expanded arch’s anchorage design involves intra-maxillary anchorage, including bilateral posterior tooth mutual anchorage and adjacent tooth anchorage (Wu et al., 2019). In the M3 group, premolars and the first molar were designed to be moved buccally. However, a displacement difference between the second premolar and the aligner hindered adjacent tooth anchorage, resulting in lower actual anchorage for the second premolar. This explains the minimal buccal displacement of the second premolar in the M3 group (Figure 2C; Table 2). In contrast, the M2 group achieved adjacent tooth anchorage for both the first premolar and first molar. Consequently, M2 exhibited the lowest anchorage loss rate and highest target tooth displacement efficiency. Groups M1 and M3 had only partial adjacent tooth anchorage, resulting in lower buccal displacement compared to M2.

To achieve efficient buccal movement with minimal anchorage loss, it is advisable to use alternating movements in CAT for arch expansion. Alternating movement resembles staging support control in aligners, such as frog pattern intrusion of anterior teeth and sequenced molar distalization. (Rossini et al., 2015; Ravera et al., 2016).

To mitigate the risk of buccal gingival recession and bone dehiscence (Kraus et al., 2014) caused by oblique buccal tooth movement (Lione et al., 2021), we incorporated various types of embossments on the aligner to enhance torque control. The embossment structures in posterior teeth aimed to exert pressure on the cervical part of the crown, similar to the power ridge in anterior teeth, facilitating torque control (Simon et al., 2014; Cheng et al., 2022a). Different shapes and thicknesses of embossments were designed based on mechanical principles, considering the extent of pressure generated by interference (Cheng et al., 2022b).

While the embossment structure improved movement efficiency, it primarily resulted in excessive tilting movements due to uncontrolled torque. Unfortunately, the embossment failed to provide beneficial torque control for posterior teeth, similar to the power ridge on anterior teeth (Cheng et al., 2022b). This failure could be attributed to the aligner material’s limited force generation, unable to support buccal bodily movement of posterior teeth adequately (Zhou and Guo, 2020). Consequently, the aligner’s support on the buccal aspect of posterior crowns was insufficient (Elkholy et al., 2015), leading to pronounced tilting movements instead of desired torque control through embossment.

Compared to cuboid and ball structures, cylindrical embossments caused more noticeable tilting movements. Conversely, ball embossments had a smaller impact on crown movement efficiency compared to cylindrical and cuboid embossments, likely due to differences in the contact area between the embossment and crown. The domed structure of the cylinder provided more resistance to compression, similar to the preference for domed structures in buildings, resulting in a more significant effect on first molar movement (Paris et al., 2020). For the first premolar, both cylinder and cuboid embossments exhibited similar performance, possibly due to differences in crown curvature affecting the distribution of resistance. Further investigation based on mechanical knowledge is required to explore this aspect.

Furthermore, torque compensation could enhance the torque control in buccal movement of the posterior teeth. We found that the tendency for tilting movement was gradually controlled as the degree of torque compensation increased, as shown in Figure 6B. Based on the principle of action and reaction, the gradual homogenization of surface stress distribution on the PDL indicates an increasingly improved tendency towards tilting movement of teeth with increasing compensation angle, as shown in Figure 8A. According to the prediction by the regression model, when the compensation angle was approximately 13°, bodily movement of the first premolar was achieved; however, the crown point movement efficiency decreased to 5.6%, as shown in Figures 6D, E. Although these findings are only based on simulations, it was difficult to achieve both good torque control and good movement efficiency during arch expansion through CAT. This may be attributable to the limited force exerted by the aligner (Zhou and Guo, 2020).

For arch expansion, we should first observe the relationship between teeth and alveolar bone and then estimate the extent of movement of the crown and root while maintaining appropriate torque compensation. In fact, in many cases of dental arch narrowing, the posterior teeth are inclined to the palatal side, which necessitates buccal inclination of the teeth rather than bodily movement. In addition, in cases of excessive buccal inclination of posterior teeth, when the compensation limit has been reached and the use of aligners for arch expansion may not be conducive for the root-bone relationship, micro-implant-assisted rapid palatal expansion (de Oliveira et al., 2021) or surgically assisted rapid maxillary expansion (Lin et al., 2022) should be considered. In addition, the attachment of CAT was not designed in our study because aligner wrapping around the buccolingual aspect of the crown provides better force for buccal-palatal movement. A related previous study found no significant effect of attachment on the efficiency of arch expansion (Zhao et al., 2017). However, based on clinical experience, attachments can increase the fit of the aligner and thus allow persistent orthodontic forces; therefore, we recommend their routine application in clinical practice.

FEA is one of the most suitable methods for studying the biomechanics of aligners. Our study provides valuable reference information for arch expansion with aligners in the clinic; however, some limitations cannot be ignored. Although FEA involves transient biomechanical simulation, it cannot fully simulate the real oral environment and material properties. Moreover, FEA is incapable of directly observing the precise biological remodeling of alveolar bone. In this experiment, the direction of periodontal tissue remodeling can only be inferred through stress distribution, thus constituting a limitation of the present study. Further improvements in parameters and optimization of methods are required to create a more realistic simulation of clinical situations through FEA.

## 5 Conclusion

Improvements of tooth movement efficiency and torque control during arch expansion with clear aligners were analyzed and investigated. With the limitations of this study, the following conclusions were drawn.

- During arch expansion with CAT, alternating movement could result in higher movement efficiency with lower anchorage loss as compared with whole movement.
- The embossment structure could improve movement efficiency during arch expansion with CAT, but it is insufficient to produce torque control; thus, exhibiting a more marked tilting movement.
- Embossment of the cylindrical structure could result in a higher movement efficiency than that with cuboid and ball structures.
- Torque compensation could enhance torque control. The torque per millimeter of the first premolar decreased by 0.26°/mm for approximately every 1° increase in compensation.

## Data Availability

The original contributions presented in the study are included in the article/Supplementary Material, further inquiries can be directed to the corresponding author.
