# Supplementary figures and images for: Improvements of tooth movement efficiency and torque control in expanding the arch with clear aligners: a finite element analysis
Source: Front Bioeng Biotechnol. 2023 Jun 1;11:1120535. doi: 10.3389/fbioe.2023.1120535 (PMC10267454; doi:10.3389/fbioe.2023.1120535)

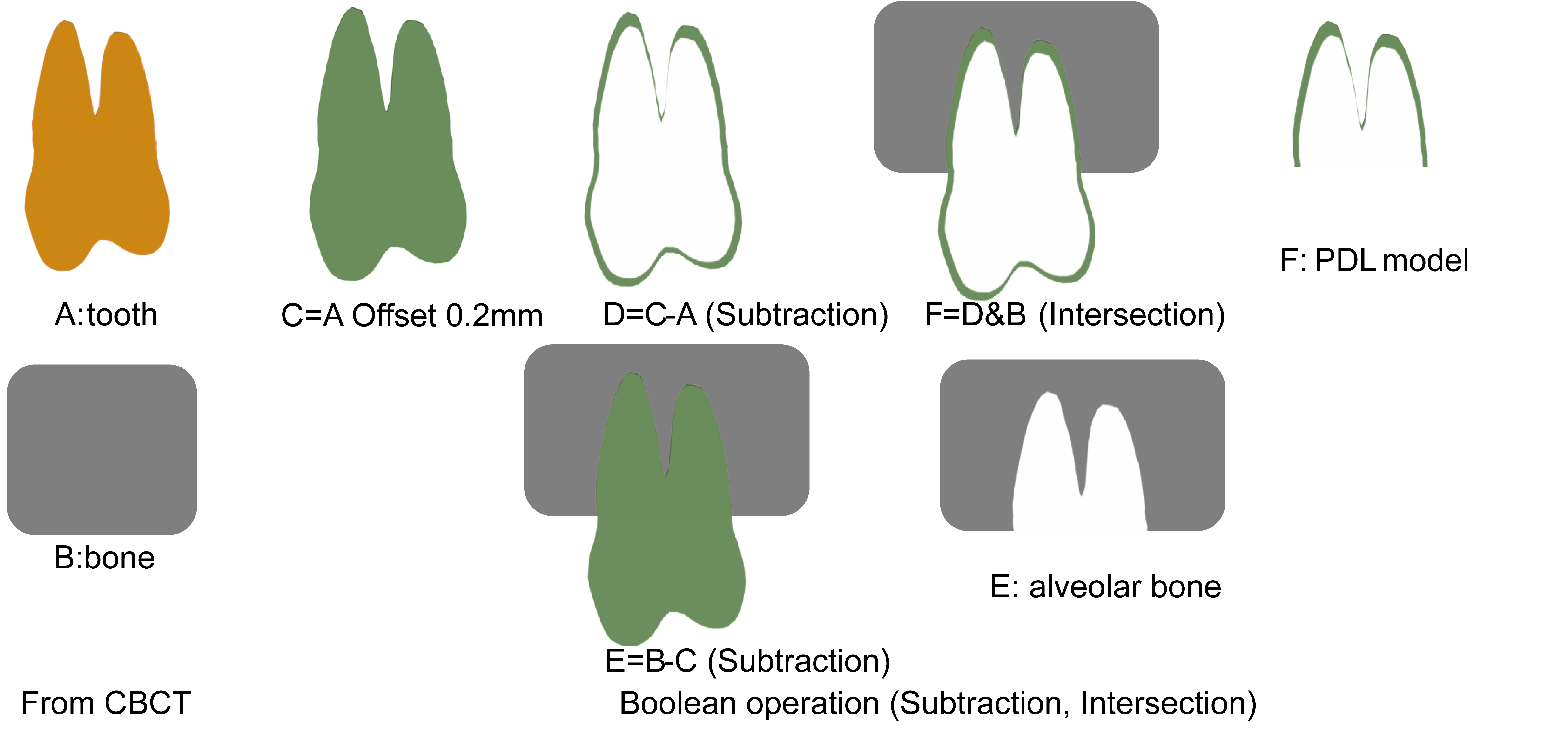

Supplement: Supplementary file 3 [file Image1.TIF]
